# Impact of ‘Enhanced’ Intermediate Care Integrating Acute, Primary and Community Care and the Voluntary Sector in Torbay and South Devon, UK

**DOI:** 10.5334/ijic.5665

**Published:** 2022-02-14

**Authors:** Julian Elston, Felix Gradinger, Sheena Asthana, Matthew Fox, Louise Dawson, Dawn Butler, Richard Byng

**Affiliations:** 1 Community and Primary Care Research Group (Faculty of Health), Plymouth University, UK; 2 Plymouth Institute of Health and Care Research (PIHR), Plymouth University, UK; 3 Torbay and South Devon NHS Foundation Trust (TSDFT), Torquay, UK; 4 General Practitioner, Barton Practice, Dawlish, UK

**Keywords:** intermediate care, multi-disciplinary teams, person-centred care, voluntary sector, early supported discharge, admission avoidance

## Abstract

**Introduction:**

Intermediate care (IC) was redesigned to manage more complex, older patients in the community, avoid admissions and facilitate earlier hospital discharge. The service was ‘enhanced’ by employing GPs, pharmacists and the voluntary sector to be part of a daily interdisciplinary team meeting, working alongside social workers and community staff (the traditional model).

**Methods:**

A controlled before-and-after study, using mixed methods and a nested case study. Enhanced IC in one locality (Coastal) is compared with four other localities where IC was not enhanced until the following year (controls), using system-wide performance data (N = 4,048) together with *ad hoc* data collected on referral-type, staff inputs and patient experience (N = 72).

**Results:**

Coastal showed statistically significant increase in EIC referrals to 11.6% (95%CI: 10.8%–12.4%), with a growing proportion from GPs (2.9%, 95%CI: 2.5%–3.3%); more people being cared for at home (10.5%, 95%CI: 9.8%–11.2%), shorter episode lengths (9.0 days, CI 95%: 7.6–10.4 days) and lower bed-day rates in ≥70 year-olds (0.17, 95%CI: 0.179–0.161). The nested case study showed medical, pharmacist and voluntary sector input into cases, a more holistic, coordinated service focused on patient priorities and reduced acute hospital admissions (5.5%).

**Discussion and conclusion:**

Enhancing IC through greater acute, primary care and voluntary sector integration can lead to more complex, older patients being managed in the community, with modest impacts on service efficiency, system activity, and notional costs off-set by perceived benefits.

## Introduction

Health care systems worldwide are striving for the ‘triple aim’ of better health for their populations, improved experience of care for patients and lower system costs [1,2]. In the United Kingdom from 2001 onwards [3], intermediate care has been seen as a way of preventing admission to hospital and supporting patients who are ready to leave hospital yet require further support at home. Intermediate Care (IC) or Transition(al) Care as it is known in some countries [4] is organised around ten service elements [5,6] which facilitate patient care and transitions between acute, community and home settings, depending on their changing support needs.

Typically, IC provides a range of integrated service models providing home-based and bed-based care, sometimes with reablement and crisis response support for up to six weeks. These aim to support timely discharge from hospital, promote faster recovery from illness, maximise independent living, and to prevent unnecessary hospital admission and premature admission to long-term residential care [7,8].

However, reviews of services promoting early discharge and avoiding inappropriate hospital (re)admissions reveal a wide range of service configurations [2]. Within the UK alone, national audits of intermediate care have repeatedly shown significant variation in service provision across the country [9], in part due to NHS reconfiguration and policy change and financial and professional barriers between health and social care services [9,10]. More recently, National Institute for Health and Care Excellence (NICE) guidance has sought to standardise key elements of delivery considered important to quality and effectiveness [11].

Systematic reviews suggest that IC-type services are effective for delivering early supported discharge in older people or for people with stroke, chronic obstructive pulmonary disease (COPD), congestive heart failure (CHF) and cognitive impairment [2,12,13]. A range of neutral or positive patient-related and health service outcomes (mortality, reduced length of care, re-admission rates, functional abilities, psycho-social well-being, overall patient and carer’s health) are reported compared to usual care, but not consistently across trials and with little evidence of cost savings. Evidence of a positive impact on admissions avoidance is similar, with increased patient satisfaction and chance of living at home at six months, but mixed results on reducing length of stay, days of care provided and cost [13,14]. This includes the sole UK-based study that focuses on older people referred by their GP [15,16,17]. There are very few UK studies reporting on the impact on home-based IC services for older people in relation to both early support discharge and admission avoidance, and these are non-randomised [18].

One of the challenges when synthesising the evidence is the heterogeneity of IC service design, team composition and delivery locations (home, community hospital, care homes). This makes it difficult to expect similar results when generalising findings to local contexts [6,19]. Nevertheless, a recent secondary analysis of national IC datasets suggests better patient outcomes may be associated with interdisciplinary team working, and stressed the need to understand how outcomes relate to team-level and process factors [20].

Interdisciplinary working has become increasingly prominent in national policy [7,21], including the NHS Long-Term Plan [22]. This promotes new service delivery models that emphasise inter-professional teams and closer working between primary, community and secondary care services as well as with the voluntary sector. It also promotes person-centred care [22,23,24]; care that recognises patient values and preferences, ensures choice and shares control. It is assumed that this strategy will reduce demand on acute care from an ageing population [10,22,25,26,27], provide a more effective and efficient service for patients with complex health needs, whilst improving their experience of care [28,29].

Against this background, Torbay and South Devon NHS Foundation Trust (TSDFT), an Integrated Care Organisation in South West England, has committed to enhancing its IC services through multi-professional coordination of more complex, high-intensity patients in the community, in people’s homes and in short-term care placements.

TSDFT provides acute and community services to approximately 286,000 people, mainly resident in six market and coastal towns across Torbay and South Devon. It has a high proportion of older people compared to England (22% residents are over 70 years) and pockets of deprivation, particularly in Torbay [30]. It is well-known for integrated care, following the creation of the Torbay Care Trust in 2005, and subsequent merger with the acute NHS Trust in October 2015 [31]. The Trust provides adult social care in two of its five localities, with pooled budgets and a risk sharing agreement with Torbay Council social services [32,33], and jointly commissions and manages health and social care teams in other localities.

The Trust’s enhanced IC model emphasised the need for a person-centred, coordinated approach to care, embodied in the strap line of ‘what matters to me, not what is the matter with me’ and delivering ‘care closer to home’ [34]. In 2016, it embarked on a phased roll-out of Enhanced Intermediate Care (EIC), starting in Coastal locality where it enhanced its Multi-Disciplinary Team (MDT) to include a wider range of professionals (a GP, pharmacist and voluntary sector Well-being Coordinator), whilst reducing community hospital beds. The remaining four localities, characterised by a more traditional IC model, involving partnerships between health and social care, sought to implement EIC one year later. Although there were some differences in service configuration between the localities [35], the staged implementation offered an opportunity for a controlled before-and-after study.

The aim of this paper is to compare the Coastal EIC service with the IC services in four other localities with respect to service activity. A nested case study in Coastal locality also assesses referral-type, staff inputs to care, patient experience and the perceived service benefits (cost off-set study). The evidence is used to make inferences about what might have made the most difference.

## Methods

### Study design

A controlled before-and-after study, comparing the impact of EIC in Coastal locality before and after implementation in March 2016 (preceded by a 6-month pilot) and with four control localities over the same time-period, where EIC was introduced a year later.

### Controls

Four other localities where EIC was implemented in April 2017, although only partially due to national GP and pharmacist workforce shortages and a less active voluntary sector hindering recruitment.

### Data sources and measurement

**Figure 1:**
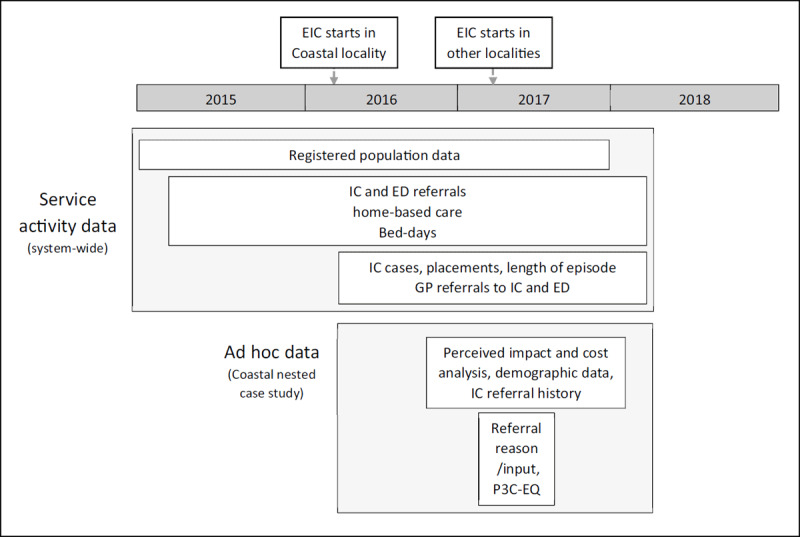
Data collection and time frames.

The evaluation drew on existing system-wide service activity data either before, during and after EIC implementation, and *ad hoc* data collected as part of a nested study in Coastal locality (see ***Figure 1***).

#### Service activity data

Number of monthly and yearly referrals to EIC/IC, source of referral, percentage and numbers placed at home or in EIC/IC beds, length of episode of care in days (aggregated to their month of discharge), and GP referrals to ED by locality between 1 April to 31 March (Financial Year) 2015–16 and 2017–18, hereafter denoted as 2015 and 2017 respectively for ease of reading.

Emergency department (ED) attendance and admissions included out-of-area cases. Bed-days were calculated by summing the number of referrals by their length of stay for the financial year.

All rates were calculated using registered GP practice populations ≥70 years aggregated to locality level for the respective year (2015, 2016, 2017) as denominators. As two GP practices ‘moved’ during 2016, these populations were allocated to their new localities, apportioned by the time spent in each.

#### Ad hoc data collection

Additional data collected for the nested case study was as follows:

*Perceived impact*: All cases referred to Coastal EIC between 30 August 2016 and 30 January 2018 (17 months) were reviewed within a week of patient discharge to assess the impact of EIC in preventing other service interventions. This assessment was made by the EIC Lead, after reviewing the patients’ electronic record, and then recorded in Excel® along with age, sex, and referral route, extracted from the Trust’s IT system. Records with missing data were excluded from the analysis (84/1031).

#### Cost off-set analysis

*Cost off-set analysis*: Interventions perceived to have been prevented by EIC were costed using national reference sources for acute and primary care as well as supporting research, with estimates older than 2016–17 adjusted for inflation based on the UK consumer price index, and rounded to the nearest pound (see ***Table 1***) [37,38,39,40,41]. The EIC unit costs per referral was estimated by summing weighted national reference costs for IC crisis response, home and bed-based care services. National unit IC costs include costs for nurse, therapist, admin staff and social worker time and some GP and pharmacist time (as UK models vary), but not for a Well-being Coordinator. All costs were multiplied by the annualised number of perceived prevented service use for each category, summed and then off-set by the estimated cost of IC multiplied by the annualised number of EIC referrals.

**Table 1:** Estimated unit costs of service use perceived to have been prevented by EIC.


INTERVENTIONS PERCEIVED TO HAVE BEEN PREVENTED	UNIT COST

Acute hospital admission [37]	£ 1,590

Emergency Department attendance [37]	£ 148

Pro-active hospital discharge [37]	£ 313

Emergency & 999 call [37]	£ 7

Out-of-hours GP visit [38]	£72

GP telephone consultation [39]	£14

Residential/nursing home admission^α^[39]	£155

Adult Social care visit (1 hour) [39]	£40

Out-of-hours nursing visit [40]	£31

Community hospital admission [41]	£140

Intermediate care^β^ [37]	£131


Key: GP = general practitioner. α: Based on the NHS contribution to a one-day admission, so it is a conservative estimate. β: Estimated by summing weighted national reference costs for crisis response, home and bed-based care services.

*Referral-type, service inputs and patient experience*: Over a six-week period (6 February 2017 to 17 March 2017) staff recorded the source, professional and service inputs for each referral up to discharge (n = 72), as part of a service evaluation. Those patients considered by staff to be able to understand or respond to a patient experience questionnaire (n = 23, 31.9%) were telephoned by a researcher or member of staff within two weeks of discharge and, after explaining about the evaluation and seeking their consent to participate, were administered the Person-Centred Co-ordinated Care Experience Questionnaire (P3C-EQ) [42]. The P3C-EQ has 11 questions which are ranked from 0 (not at all), 1 (to some extent), 2 (more often than not) and 3 (always). Domains cover important elements of EIC and the new care model such as ‘telling your story once’, understanding ‘what matters to me, not what is the matter with me’, improving self-management and developing a strengths-based approach to care.

### Bias

To minimise recall and social desirability bias, perceived impact data and PC3-EQ questionnaires were recorded and administered respectively within two weeks of discharge by senior staff not directly involved in their care.

System activity data were co-interpreted by the researchers and senior managers from all localities, which provided contextual information relating to differences in data collection and service provision. This was supplemented by in-field observations and contextual knowledge [43] – the two main authors have been working as Researchers-in-Residence in TSDFT system since March 2016 [44], attending MDTs and IC development events and management meetings.

### Data analysis

*Statistical analysis*: Calculation of 95% confidence intervals (CIs) and comparison of referral rates were based on the Poisson distribution, using the mean number of occurrences per unit time (month or year). Differences in the mean of means between localities used the T distribution to test for differences (df(12–1) = 11, 95% = 2.201), and for rates the normal distribution using a continuity correction [45]. All analyses were undertaken in Microsoft™ Excel.

### Ethical consent

Individual consent was obtained from IC staff for observational work of MDTs, events and meetings. Ethical approval to use evaluation data as a secondary data source for research purposes was granted through ‘Proportionate Review’ by the NHS Health Research Authority (Research Ethics Committee reference: 17/LO/1745; Protocol number: PSMD-208147-SA-FG-034; Integrated Research Application System project ID: 208147).

## Findings

### Comparative description of intervention in Coastal versus control sites

Across TSDFT as a whole, the IC service involved the deployment of MDTs, the use of data sharing agreements (IC team members have read-access to health and social care records) and referrals from acute and community hospitals (except Torquay locality, which does not have one). However, Coastal locality differed from its neighbours in the extent to which it has embedded multi-professional working (see ***Table 2***), hence the description of an Enhanced IC service (EIC). In this locality, the MDT comprises general practitioners (GPs) (with read and write-access to all Coastal GP records), pharmacists, and voluntary sector Well-being Coordinators in addition to the community matrons, community nurses, occupational and physiotherapists, social workers, mental health liaison staff and health and social care co-ordinators found in other localities (with external GP input requested when needed). EIC staff worked flexibly, sometimes blurring roles, in order to respond promptly to people in crisis.

**Table 2:** Summary of the population size, level of service integration and degree of implementation of the new care model across all five localities between 2016 and 2017.


DEMOGRAPHICS AND CHARACTERISTICS OF EIC BY LOCALITY	2015					2016				

	COASTAL	MOOR TO SEA	NEWTON ABBOTT	PAIGNTON & BRIXHAM	TORQUAY	COASTAL	MOOR TO SEA	NEWTON ABBOTT	PAIGNTON & BRIXHAM	TORQUAY

Area description	Towns/rural	Towns/rural	Towns	Urban/sub-urban	Urban/sub-urban	Town/rural	Towns/rural	Towns	Urban/sub-urban	Urban/sub-urban

Registered GP population size ≥70 years	7,289	6,179	11,170	14,329	11,110	7,589	6,434	11,697	14,976	11,510

Proportion of the population ≥70 years	20.4%	17.1%	16.6%	19.6%	15.4%	21.1%	17.7%	17.1%	20.3%	15.9%

Joint management of HSC team	**Yes**	**Yes**	**Yes**	**Yes** ^α^	**Yes** ^α^	**Yes**	**Yes**	**Yes**	**Yes** ^α^ ^α^	**Yes** ^α^

Joint budgets for HSC team^β^	**Yes**	**Yes**	**Yes**	**Yes** ^β^	**Yes** ^β^	**Yes**	**Yes**	**Yes**	**Yes** ^β^ ^β^	**Yes** ^β^

Single daily MDT	**Yes**	No	No	No	No	**Yes**	**Yes**	**Yes**	**Yes – two**	**Yes – two**

Single referral process/point of contact^γ^	No^γ^	No^γ^	No^γ^	**Yes**	**Yes**	No^γ^^γ^	No^γ^	No^γ^	**Yes**	**Yes**

Integrated health and social care IT records^δ^	No	No	No	**Yes**	**Yes**	No	No	No	**Yes**	**Yes**

Write access to GP records^ɛ^	**Yes**	No	No	**No**	**No**	**Yes**	**No**	**No**	**No**	**No**

Heath and social care coordinator	**Yes**	**Yes**	**Yes**	**Yes**	**Yes**	**Yes**	**Yes**	**Yes**	**Yes**	**Yes**

Social worker input daily into MDT	**Yes**	**Yes**	**Yes**	**Yes**	**Yes**	**Yes**	**Yes**	**Yes**	**Yes**	**Yes**

GP input daily into MDT	**Yes**	No	No	No	No	**Yes**	**Yes** ^ζ^	No	**Yes** ^θ^	No

Pharmacy input daily into MDT	**Yes** ^ι^	No	No	No	No	**Yes**	No	No	No	Yes

Voluntary sector input daily into MDT	**Yes** ^κ^	No	No	No	No	**Yes**	No	No	No	No

Community MH input daily into MDT	**Yes** ^λ^	No	No	**Yes**	**Yes**	**Yes**	No	No	**Yes**	**Yes**

Other input daily into MDT	**No**	No	No	No	No	**No**	**Yes**	**Yes** ^η^	No	No

Community hospital-bed reductions/closures	**Yes**	No	No	No	NA	**Yes**	**Yes**	**Yes**	**Yes**	NA


KEY: EIC = Enhanced Intermediate Care; GP = General practice/practitioner; HSC = Health and social care; MDT = Multi-disciplinary meeting; IT = Information Technology. *Note*: α Since 2002 and then formerly in 2005 with the creation of Torbay Care Trust β Aligned budgets while building towards Section 75 agreements γ Social services and health service have a different Single Point Of Contact (SPOC) δ Integrated community health and social care notes recorded on PARIS ɛ Write access but only through the attending GP (not the whole team). Other areas had read access only. ζ Visimeet audio-visual system introduced in 2018 η Paramedic started in 2018. Community matron only twice a week. Θ Attends MDT twice a week in the morning (2 P.A.s) ι Attends MDT four times a week in the morning (4 P.A.s) κ Two alternating Well-being Coordinators five times a week (5 P.A.s) λ Community Mental Health Nurse once a week.

The MDT met five days a week to triage, discuss and plan the care for approximately 30 high-risk or newly discharged patients each day, with the core team linking and providing pro-active care at other times. It also linked patients into local voluntary groups, activities and resources as part of a wider social prescribing offer [36], if considered beneficial. This offer sometimes involved Well-being Coordinators developing plans with patients using goal-setting tools, supported by 12 weeks of coaching and practical and emotional support, with the aim of improving their independence and well-being.

**Figure 2:**
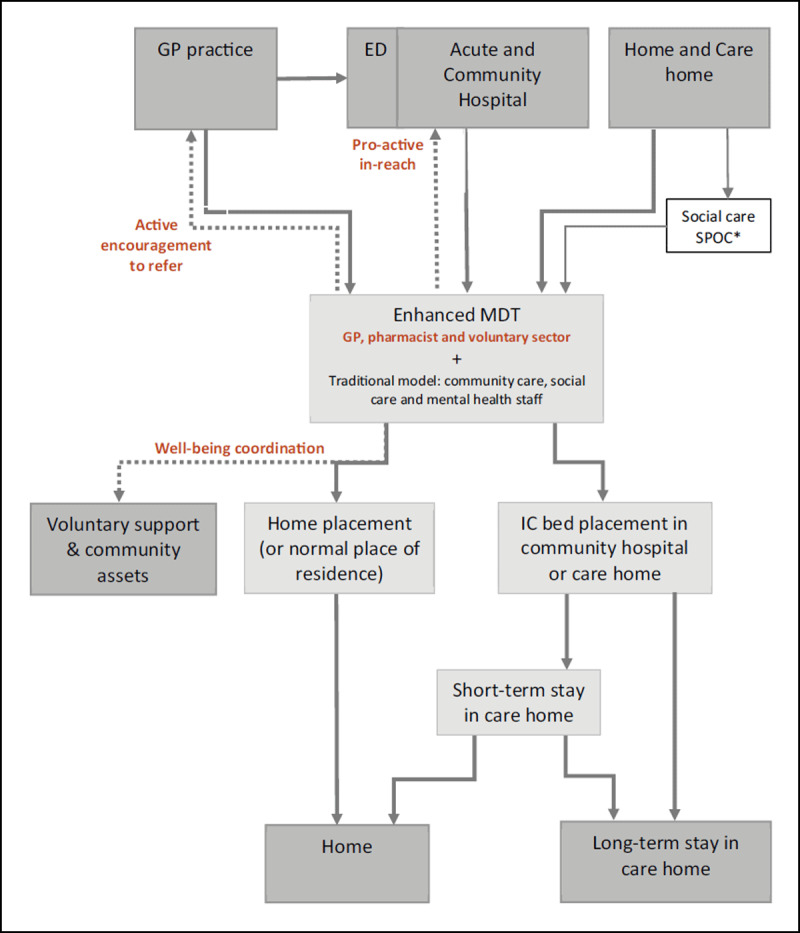
Intermediate care referral pathways, including the ‘enhanced’ pathway in Coastal locality. Key: The enhanced elements of IC are marked in bold, brown text and new pathways shown with dotted lines. The solid lines are pathways that are present in both EIC and IC. ED is emergency department. * South Devon localities also receive referrals from social care’s single point of contact (SPOC) service. Torbay localities have a combined health and social care SPOC.

The EIC team was co-located within the Teignmouth Health & Well-being hub (a former community hospital), where health and social care staff are jointly managed, but did not have a pooled budget. The team pro-actively engaged with the acute hospital prior to patient discharge and actively encourage local GPs and the ambulance service (SWAST) to refer deteriorating older patients to them rather than to the emergency department. ***Figure 2*** shows how Coastal EIC differed to IC pathways in other localities.

### Comparison of service activity in COASTAL versus control sites

***Table 3*** shows that two years after the introduction of EIC in Coastal, it had moved from being the locality with the lowest referral rate to that with the highest, reaching 11.6% of the ≥70 year-old population in 2017. Rates in other localities plateaued in 2017. There was almost a two-fold variation in referral rates between Coastal and the locality with the lowest rate, differences that were statistically significant.

**Table 3:** Population size (≥70 years), rates per ≥70 year-old population and percentage change in rates by year.


LOCALITY	POPULATION SIZE				EIC REFERRAL RATES (95% CI)				% CHANGE IN RATES	
		
	2015	2016	2017		2015	2016	2017		2015 to 2016	2016 to 2017

Coastal	7,104	7,289	7,589		4.3% (3.8%–4.7%)	7.0% (6.4%–7.6%)	11.6% (10.8%–12.4%)*		64.9%	65.6%

Moor to Sea	8,605	6,179	6,434		7.7% (7.1%–8.3%)	12.0% (11.1%–12.8%)	8.2% (7.5%–8.9%)		55.2%	–31.3%

Newton Abbot	8,265	11,170	11,697		6.5% (5.9%–7.0%)	8.5% (8.0%–9.0%)	10.3% (9.7%–10.8%)		30.8%	20.7%

Paignton and Brixham	14,010	14,329	14,976		6.7% (6.3%–7.2%)	6.6% (6.2%–7.0%)	6.6% (6.2%–7.0%)		–2.2%	0.7%

Torquay	10,963	11,110	11,510		6.3% (5.8%–6.8%)	8.1% (7.6%–8.7%)	7.8% (7.3%–8.3%)		29.4%	–3.9%

Total (not incl. Coastal)	41,843	42,788	44,617		6.8% (6.5%–7.0%)	8.3% (8.0%–8.5%)	8.1% (7.9%–8.4%)		22.0%	–1.8%

Total	48,947	50,077	52,206		6.4% (6.2%–6.6%)	8.1% (7.8%–8.3%)	8.6% (8.4%–8.9%)		26.2%	6.7%


Key: EIC = Enhanced Intermediate Care; * = statistically significant (p ≤ 0.05), compared to other localities and Total (not incl. Coastal) within year. *Note*: Moor to Sea referrals were recorded differently after 2016 bringing it into line with other areas, possibly explaining the fall in cases. The locality boundary also changed but this was adjusted for.

Coastal had the shortest average monthly length of episode for EIC cases by at least 5 to 6 days compared with other localities (***Table 4***). These differences were statistically significant over both years. By 2017 it had the lowest percentage of cases placed in an IC bed, and those placed had the shortest average monthly length of episode and stay of all localities, although this grew by 16% in 2017. The average monthly length of episode and stay (data not shown) for home-based care was also the shortest of all localities by at least 4 to 5 days, a statistically significant difference.

**Table 4:** Yearly mean of average monthly length of episode (LOE) (days) for intermediate care, patients placed in IC beds and home-placed patients for 2016 and 2017.


	EIC CASES – AV. MONTHLY LOE (DAYS) (95% CI)			% EIC CASES PLACED (95% CI)			IC PLACEMENTS – AV. MONTHLY LOE (DAYS) (95% CI)			IC HOME-BASED CARE – AV. MONTHLY LOE (DAYS) (95% CI)	
			
LOCALITY	2016	2017		2016	2017		2016	2017		2016	2017

Coastal	7.9 (6.8–8.9)*	9.0 (7.6–10.4)*		9.4% (5.6%–13.2%)	8.7% (7.2%–10.2%)		19.1 (15.2–23.0)	22.2 (17.5–26.9)		6.6 (5.9–7.3)*	7.8 (6.5–9.2)*

Moor to Sea	13.0 (11.6–14.4)	15.1 (14.0–16.1)		9.5% (5.5%–13.6%)	20.1% (16.3%–23.9%)		26.9 (20.3–33.4)	27.1 (23.1–31.1)		11.5 (10.5–12.6)	12.2 (10.6–13.9)

Newton Abbot	42.3 (31.7–52.9)	20.1 (17.7–22.5)		5.5% (3.9%–7.1%)	9.1% (6.0%–12.2%)		63.7 (53.7–73.8)	33.8 (21.3–46.2)		40.9 (29.7–52.1)	18.5 (16.8–20.2)

Paignton and Brixham	41.2 (36.9–45.5)	33.6 (28.3–38.9)		29.3% (23.9%–34.7%)	40.5% (33.8%–47.2%)		37.3 (29.6–45.0)	29.3 (24.5–34.0)		41.4 (35.9–46.8)	36.0 (28.6–43.4)

Torquay	21.7 (19.6–23.8)	20.8 (18.9–22.6)		41.0% (35.6%–46.3%)	47.3% (42.7%–52.0%)		24.9 (22.0–27.8)	25.0 (22.2–27.8)		18.7 (15.6–21.9)	15.7 (12.4–19.0)

Total	27.9 (24.9–30.9)	20.4 (18.9–21.8)		19.9% (17.2%–22.6%)	25.0% (23.1%–26.9%)		32.2 (28.5–35.9)	27.2 (24.2–30.2)		26.5 (22.5–30.6)	17.8 (16.1–19.6)


Key: EIC = Enhanced Intermediate Care; IC = Intermediate Care; LOE = Length of episode; Av. = average; * = statistically significant (p ≤ 0.05), compared to other localities within year.

***Table 5*** shows that Coastal moved from the locality with the lowest rates of home-base care and GP referral rates to that of the highest rates by 2017 (10.5%, 95% CI: 9.8%-11.2% and 2.9%, 95% CI: 2.5%-3.3%, respectively). These differences were statistically significant compared to other localities in that year. Over the same time GP referral rates to ED (an alternative destination to EIC for deteriorating frail, older people) were lowest in Coastal between 2016 (statistically significant) and 2017. Assuming this cohort of patients is similar across localities in relation to relative size and severity, the ratio of GP to ED referral rates gives an indication of how well EIC is preventing ED admissions. ***Table 5*** shows that Coastal had the largest ratios, reaching 0.93 in 2017 compared to 0.34 for all other localities combined. This was despite a slight, unexpected 0.2% increase in ED referral rates in 2017, which contrasted with an overall 0.2% fall elsewhere. However, these falling rates were still substantially higher than in Coastal by 2% for all other localities combined.

**Table 5:** Intermediate care home referral rates, GP referral rates to EIC and to Emergency Department (ED) for ≥70 year-olds and referral rate ratios between GP and ED for 2015 and 2017 by locality.


LOCALITY	HOME-BASE CARE REFERRAL RATES (95% CI)				GP REFERRAL RATES TO EIC (95% CI)			GP REFERRALS RATES TO ED (95% CI)			GP: ED REFERRAL RATE RATIO	
			
	2015	2016	2017		2016	2017		2016	2017		2016	2017

Coastal	3.6% (3.1%–4.0%)	6.3% (5.7%–6.9%)	10.5%* (9.8%–11.2%)		1.5% (1.2%–1.8%)	2.9%* (2.5%–3.3%)		2.9%* (2.5%–3.3%)	3.1% (2.7%–3.5%)		0.51	0.93^β^

Moor to Sea	6.9% (6.3%–7.4%)	10.8% (10.0%–11.6%)	6.5% (5.9%–7.1%)		2.1% (1.7%–2.4%)	1.9% (1.5%–2.2%)		4.2% (3.7%–4.7%)	3.7% (3.3%–4.2%)		0.49	0.50

Newton Abbot^α^	6.1% (5.5%–6.6%)	7.9% (7.4%–8.5%)	9.1% (8.5%–9.6%)		2.3% (2.0%–2.5%)	2.0% (1.7%–2.2%)		5.6% (5.1%–6.0%)	5.1% (4.7%–5.5%)		0.41	0.38

Paignton and Brixham	5.0% (4.6%–5.3%)	4.3% (3.9%–4.6%)	3.5% (3.2%–3.8%)		2.2% (1.9%–2.4%)	1.7% (1.5%–1.9%)		5.0% (4.7%–5.4%)	4.6% (4.3%–5.0%)		0.43	0.37

Torquay	3.7% (3.3%–4.0%)	4.4% (4.0%–4.8%)	3.7% (3.4%–4.1%)		1.9% (1.6%–2.1%)	1.5% (1.3%–1.7%)		6.5% (6.0%–6.9%)	6.7% (6.2%–7.2%)		0.29	0.22

Total (not Coastal)	5.2% (5.0%–5.4%)	6.2% (6.0%–6.4%)	5.4% (5.2%–5.7%)		2.1% (2.0%–2.2%)	1.7% (1.6%–1.9%)		5.4% (5.2%–5.6%)	5.2% (4.9%–5.4%)		0.39	0.34

Total	5.0% (4.8%–5.2%)	6.2% (6.0%–6.4%)	6.1% (5.9%–6.4%)		2.0% (1.9%–2.1%)	1.9% (1.8%–2.0%)		5.1% (4.9%–5.3%)	4.9% (4.7%–5.0%)		0.40	0.39


Key: EIC = Enhanced Intermediate Care; GP = General practice; ED = Emergency department; GP: ED referral rate ratio = the rate of GP referrals to EIC divided by the rate of GP referrals direct to ED; * = statistically significant (p ≤ 0.05) compared to other localities and Total (not incl. Coastal) within year, using continuity correction. *Note*: ^α^ Included part of Moor to Sea in 2016, although this was adjusted for. ^β^ A high relative ratio persisted in 2018 with ratios in other localities remaining similar.

The results suggest that overall the Coastal locality was managing IC patients differently to other localities with relatively less IC bed-based placements and shorter episodes of both bed-based and home-based care. This enabled more individuals to be seen as increasing referral rates were matched by throughput.

**Figure 3:**
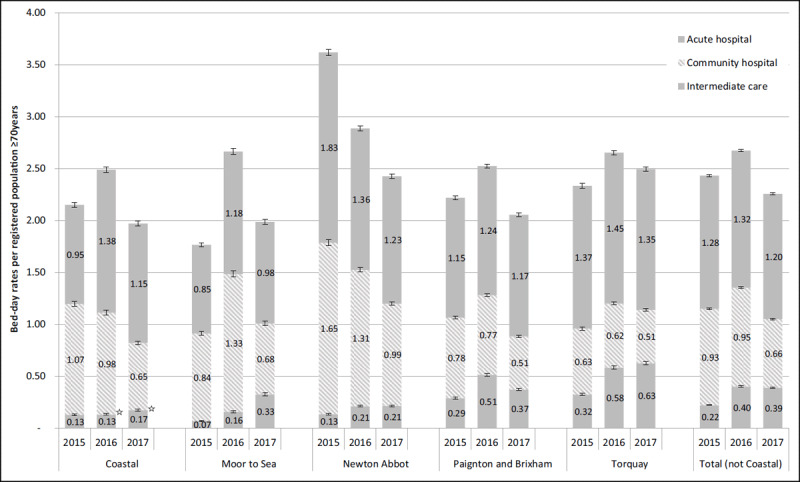
Trends in bed-day rates for intermediate care beds, community and acute hospital beds for ≥70 year-olds by locality between 2015 and 2017. Key: ☆ = p ≥ 0.05 compared to other localities within year.

***Figure 3*** shows bed-day rates for EIC and the community and acute hospitals for ≥70 year-olds by locality between 2015 and 2017. Coastal had the lowest total bed-day rates (1.97) compared with other localities for 2016 and 2017 (although not statistically significant) and was 8.4% lower in 2017 than prior to EIC implementation. In relation to EIC, rates generally increased for all localities by 2017, but for Coastal this increase was smaller despite it having significantly higher referral rates than other localities. This can be accounted for by the statistically shorter average length of episode and stay (data not shown) and statistically significant higher rates of home bed-days than other localities (see ***Tables 4*** and ***5***), suggesting efficiency gains from faster turnarounds. Community hospital bed-day rates fell in all localities, with the largest percentage falls from 2015 in Coastal (39.5%) and Newton Abbot (40.3%) localities. Acute bed-day rates also fell overall between 2015, but saw a mixed picture with some localities increasing slightly and other decreasing. Nevertheless, by 2017 Coastal still had the second lowest rate compared to other localities.

### Nested case study

#### Characteristics of Coastal EIC patients and inputs

In the 17-month nested study during which referral records were reviewed, there were 947 Coastal referrals, relating to 642 people. This equated to 12.9 per week.

Over half of referrals were female (60.6%). 70.9% were over 80 years old; 19.9% were aged 70–79 years. The average age was 83.1 years and median of 85 years (range 33–105 years). The average number of referrals per person was 1.5, but positively skewed (range 1–7, median 1). Of the 642 patients, 69.9% were referred once (n = 449), 18.8% twice (n = 242), 7.2% trice (n = 46) and 4% four or more times (n = 26).

In the six-week nested study, there were 72 referrals, a third from GPs (37.5%), 25.0% from community services and 16.7% from the acute hospital. Two-thirds of referrals were for poor mobility or falls (40.3% and 29.2% respectively). The rest covered a range of: medical (dementia, UTIs, other) (15.3%); environmental issues, and; transitions between services. GP referrals included a slightly greater proportion of medical and mental health issues (22.2%).

Nearly half of all referrals required input from a physiotherapist (48.6%), occupational therapist (44.4%), community nurse (44.4%) or social worker (44.4%), and a third from a community matron (34.7%). In relation to the ‘enhanced’ inputs, GPs actively contributed to just over a third (36.1%) of cases, and the pharmacist and the voluntary sector to around 1 in 7 cases (15.2% and 12.5% respectively). The rapid response and social care reablement teams inputted 23.6% and 12.5% respectively.

**Figure 4:**
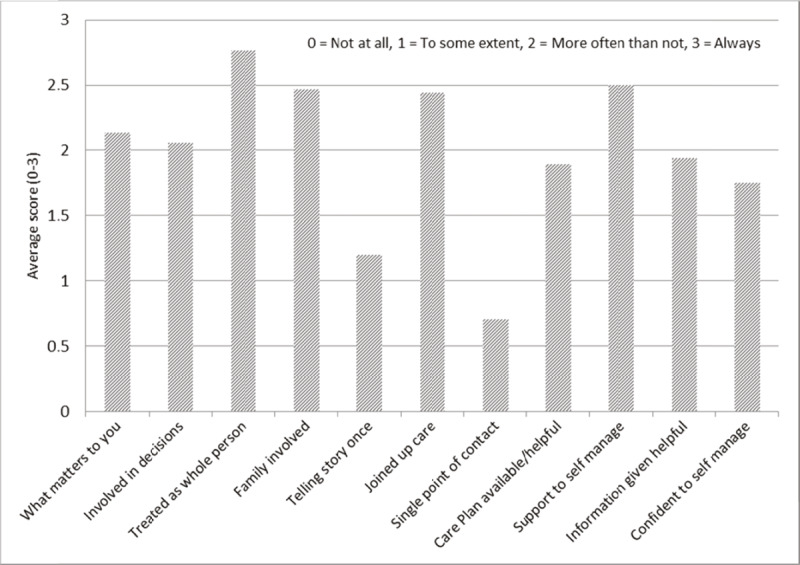
Patient experience questionnaire (P3C-EQ, N = 17), Coastal locality.

Patient experience data is shown in ***Figure 4***. Only 17/23 (73.9%) were contactable within two attempts. All agreed to participate. The P3C-EQ domains with the highest scores were ‘treating people as a whole person’, ‘supporting self management’, ‘feeling joined-up’ and ‘involving the family’. Domains relating to co-ordination were less strong: ‘care planning’, ‘single point of contact’ and ‘telling your story once’.

### Estimated impacts on health and social care costs

**Figure 5:**
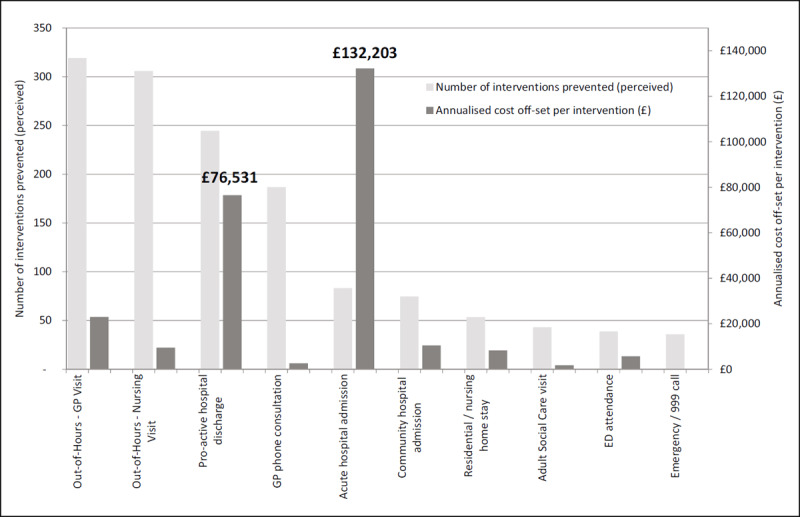
Perceived prevented impact of enhanced intermediate care and associated costs (annualised) in Coastal locality.

***Figure 5*** shows the annualised number of interventions judged to have been prevented by EIC (left-hand axis) and their annualised cost (right-hand axis). This suggests that EIC is perceived to have benefited the health care system beyond the ICO, particularly through reducing GP telephone consultations and out-of-hours visits (just under one per day each), social care visits, nursing and residential care stays and emergency calls. Within the ICO, early supported discharge showed the most benefit, greater than acute and community hospital admissions and ED attendances. In relation to the total number of ED attendances and acute admissions from Coastal, this represented a modest 2.3% (39/1,709) and 5.5% (83/1,501) of annualised number of attendances and admissions respectively in those ≥70 year-olds over the same period.

However, when this perceived activity prevented was costed, the financial benefit largely fell to the ICO, with acute hospital admission and early support discharge accounting for 77.2% of total benefit (£270,320). Off-set against the tariff cost for each referral (£131), the overall annualised ‘notional’ cost off-set of EIC was £182,970 (£193 per referral).

Impacts on bed days should be contextualised as, during the study period, capacity generally decreased as beds were removed from community (23% or 50 beds) and acute hospitals (8% or 41 beds), starting with Coastal community hospital in August 2016 (11 beds) and elsewhere in March 2017. A fall in rates was thus expected across all localities by 2017, not least because the reduction in beds was greater than the rate of beds unoccupied, and localities were encouraged to care for more people at home (home bed-days).

## Discussion

### Outcomes of EIC

Enhancing Coastal locality’s IC team was associated with statistically significant increases in IC referral rates, home-based care rates and GP referral rates to EIC, and shorter average length of episode and lower bed-day rates compared to other localities. In addition, the nested case study also suggests that EIC is likely to have contributed to reducing activity across the health and social care system, particularly in primary care, but also in acute care. Although the perceived prevention of reduced ED admissions and increased pro-active hospital discharges were relatively small numerically, they accounted for most of the financial benefit of EIC. Coastal also had the lowest GP referral rate to ED, possibly due to the higher rate of GPs referring deteriorating, older people to EIC (actively encouraged by the team), a conclusion also supported by its markedly different rate ratio compared to other localities. In addition, the nested case study showed that nearly one in six referrals had a medical issue, with a third requiring medical input and a seventh pharmacist input. This also suggests that the EIC team was managing more complex patients often with medical as well as therapeutic needs [31]. Patients with mild to moderate frailty reported receiving moderate to high levels of person-centred coordinated care, with potential for improvement on care planning, ‘telling your story once’ and accessing care through a single point of contact.

There was also evidence of voluntary sector input in the MDT. Although the nested study did not evaluate this, the authors were also involved in a concurrent before and after study assessing the impact of Well-being Coordination in the Coastal locality, including EIC referrals. This showed a positive impact on patient well-being, patient activation and independence [46], and noted the bio-psycho-social complexity of many of those referred to EIC and the psychological, social and practical support the voluntary sector can provide i.e. informally coordinating care and connecting them to community resources. Similar benefits were reported in a pilot study in Wales that referred IC patients to a voluntary sector coordinator, albeit not located in the MDT [47].

TSDFT is known internationally for its high levels of health and social care integration [31,32]. Its IC service is already performing above average on most of the 18 NICE performance indicators, and at lower cost per service user [48]. These results suggest that enhancing integration with acute and primary care, pharmacy and the voluntary sector can lead to additional benefits for the patient and the health and social care system, supporting UK policy assumptions [21,22].

### Other factors influencing the delivery of EIC

However, it is likely that other factors, aside from those identified in ***Table 1*** contributed to the positive impact of EIC. Coastal locality was an earlier adopter of EIC, suggesting that its capability to integrate [49] was different to other localities. A case study of IC capability across TSDFT, conducted by three of the authors over the same time period, identified a combination of contextual and organisational factors at play [31]: a history of collaboration between GPs and community teams and a well-developed voluntary sector, providing a range of community support services; the smallest population, enabling all referrals to be managed within one MDT; the co-location of different professional teams enabling informal MDT working; a person-centred culture, focused on early mobilisation and independent living, and; shared clinical leadership [50], supported by a GP who was also a Locality Clinical Lead at the ICO and the CCG (as a system-wide post to support acute and primary care integration) [51]. Many of these elements are reported to be important for effective inter-professional health and social care team working [52,53,54] and integrated care systems [55].

Other potential influential factors were the configuration, capacity and use of IC and community hospital beds, which varied by locality. Although IC beds increased across all localities with EIC implementation, overall bed-capacity reduced as some community hospitals closed. It is possible, for example, that the relatively higher supply of IC beds in care homes in Paignton & Brixham and Torquay, and community hospital beds in Newton Abbott may have contributed to the higher respective bed-day rates seen, assuming localities used all available beds.

Supply-side factors may also have explained the higher levels of managing complex patients at home in Coastal, which appeared to have the lowest overall bed capacity rate (data not shown due to its reliability). This appeared to be achieved by Coastal’s strong emphasis on early mobilisation and independence which resulted in shorter length of stays and episodes in IC and community hospital beds than in other localities, effectively increasing their overall bed-capacity through efficiency gains.

### Comparison with other studies

Evidence on the effectiveness of IC, including transitional care, is mixed, albeit with a growing number of international studies suggesting IC can improve function, reduce health care utilisation and lower costs [2]. However, it is challenging to compare the impact of EIC with other IC studies due to their differences in service model and cohort, comparators (ours being IC as usual care) and reported outcomes [12,14,17]. This has resulted in efforts to agree an international definiton of IC [6], which TSDFT’s IC services meet. Although other studies have found a correlation between community-based multi-professional teams, shorter length of stay and reduced costs, the breadth of professions involved was narrower [56]. Broader staff-type membership is correlated with improved outcomes for IC patients in the UK [20,48,57]. We could not identify any IC studies that included such a range of professionals in their MDTs as that in Coastal. Some IC services in Wales have piloted voluntary sector and general practice involvement in IC services, but to a lesser degree. This enhancement showed promising results, but interpretation was difficult as rates were not reported and the data was incomplete [58]. Our study did not directly assess the potential benefit of including a pharmacist in IC, however a pilot study in Fife, Scotland described a number of positive outcomes for patients although the study methodology was weak [59].

### Policy implications and further research

Thus, this study provides some evidence to support UK policy assumptions on integration [21,22]. However, demonstrating this is challenging not least as measures will need to be wider in scope and able to detect modest changes in outcomes. Research will also need to understand how context (area, organisation and team membership and dynamics) influences the efficiency and effectiveness of IC services to facilitate transferable understanding of how other sites can achieve similar impacts.

### Strengths of study

Evaluating EIC services poses significant practical and methodological challenges such as incomplete data collection and attribution [58]. As Researchers-in-Residence we used mixed methods, considered appropriate for studying complex interventions [60], to take advantage of a phased implementation. Guiding and improving existing data collection allowed us to compare impact across localities operating within the same meso-level context (our controls). Working closely with staff across locations allowed us to check our data interpretation, while support from University-based line-managers helped ensure we did not compromise our objectivity and critical judgement.

### Weaknesses of study

Interpreting routine data for evaluating integrated care is challenging [61] without randomised controls. Nevertheless, differential patterns in trends over time can help support understanding of causation when set alongside a contextual analysis. It is possible that other contextual differences could have contributed to Coastal EIC’s performance in this study. However, Coastal locality had pockets of deprivation and a degrees of rurality similar to other localities, which would suggest otherwise.

Some data was not available prior to implementation (e.g. IC placements), and there were subtle differences in coding practices across localities. Being embedded made us aware of these issues, allowing us to adjust calculations and interpretations accordingly. For example, not knowing about GP populations ‘moving’ may have unduly influenced locality rates. However, the adjusted data suggested referral rates to Newton Abbot IC in 2016 did not fall as a consequence (as practices got to know their ‘new’ IC team) as the rates fell the following year.

One of EIC/IC’s aims is to reduce informal caregiving and short and long-term residential care placements, and the resulting financial consequence for individuals and the system. However, it was not possible to obtain data on these outcomes. Neither was locality level data on delayed transfer of care or package of care available. Thus, we could not adjust the overall length of episode of care by locality accordingly, enabling us to make fairer comparisons. There was also limited data on IC re-referral rates and 30-day re-admission rates, both potential indicators of service safety. The cost off-set analysis used national rather than local IC unit cost estimates, as these were not available, potentially inflating the cost benefit of EIC. Although national audit data for 2018 (i.e. post EIC implementation) showed a higher than median cost for TSDFT’s home-based care, it was significantly lower for bed-based care [48].

Data on service inputs, person-centred care and perceived prevention of service use was only collected in Coastal locality, so there were no controls by which to compare the nested study findings, weakening attribution.

## Conclusion

Enhancing IC through increased medical, pharmaceutical and voluntary sector input, coordinated through a daily MDT, in a highly vertically and horizontally integrated system appears to increase service efficiency, reduce acute attendances and provide benefits across the care system, whilst delivering a person-centred service. However, implementation of EIC was constrained by contextual and behavioural factors in some localities. These need to be recognised and addressed by policy makers if ambitions for higher levels of horizonal and vertical integration are to be achieved more widely.
